# Plant-Based Diets in Children: Secular Trends, Health Outcomes, and a Roadmap for Urgent Practice Recommendations and Research—A Systematic Review

**DOI:** 10.3390/nu16050723

**Published:** 2024-03-01

**Authors:** Malgorzata A. Desmond, Mary S. Fewtrell, Jonathan C. K. Wells

**Affiliations:** UCL Great Ormond Street Institute of Child Health, London WC1N 1EH, UKjonathan.wells@ucl.ac.uk (J.C.K.W.)

**Keywords:** vegetarian, vegan, children, secular trend, bone health, growth, nutrient deficiencies, clinical practice

## Abstract

People are increasingly encouraged to reduce animal food consumption and shift towards plant-based diets; however, the implications for children’s health are unclear. In this narrative review of research in high-income settings, we summarize evidence on the increasing consumption of plant-based diets in children and update an earlier systematic review regarding their associations with children’s health outcomes. The evidence indicates that vegan, but not vegetarian, diets can restrict growth relative to omnivorous children and increase the risk of being stunted and underweight, although the percentage affected is relatively small. Bone mineral content is reduced in vegetarian and, in particular, vegan children, compared to omnivores. Both vegetarian and vegan children who do not use vitamin B12 supplements manifest with B12 deficiency; however, supplementation rectifies this problem. Both vegetarians and vegans have lower concentrations of 25(OH)D if unsupplemented, and lower body iron stores, but usually have normal iron metabolism markers. Both groups are at risk of iodine deficiency, and this might affect thyroid health. Children consuming a vegan diet have a more favorable lipid profile than omnivorous children; however, the results for a vegetarian diet are inconsistent and vary by outcome. Based on the same scientific evidence, national and international dietary recommendations are heterogeneous, with some countries supporting plant-based diets among infants, children, and adolescents, and others discouraging them. We offer a research roadmap, highlighting what is needed to provide adequate evidence to harmonize dietary recommendations for plant-based diets in children. A number of measures should urgently be introduced at international and national levels to improve the safety of their use in children.

## 1. Introduction

Around the world, populations are being encouraged to switch to plant-based diets in order to reduce the environmental burden linked to animal agriculture [1,2]. It has been estimated that the adverse environmental impact of a high-meat diet is four times greater than that of a vegan diet when it comes to greenhouse gas emissions and land use, almost three times higher for biodiversity, and two times higher for water use [3]. However, it is not just meat production that elevates greenhouse emissions; dairy foods rank second [4], with dairy cattle being responsible for 20% of food-related gas emissions, following beef cattle, with a value of 41% [5]. The call to reduce animal-based foods in human diets, originating from within academia [2], has recently translated into numerous national and international dietary guidelines around the world [1,6,7,8].

Consistent with such encouragement, numerous sources of data indicate that more people in high-income countries are adopting plant-based diets, including vegan and vegetarian diets. Although formal estimates are available for only a few countries through diet and lifestyle surveys [9,10], an increase in the popularity of plant-based diet keywords in Google searches [11] and market trend research also indicates a rapid rate of growth in plant-based dietary patterns in numerous high-income countries [10,12]. Beyond environmental concerns, the main reasons for these trends are concerns over animal welfare and human health [13].

Because plant-based diets restrict the intake of specific food groups, they may have particular implications for children’s health. Vegan diets do not contain any products of animal origin; they exclude meat, fish, eggs, and dairy. Vegetarian diets exclude meat and fish but include dairy and eggs. This changes the profile of micro- and macronutrient intake and may increase the risk of inadequate nutrient intake and nutrient deficiencies, including those of protein, calcium, vitamin D, iodine, zinc, iron, and omega-3 essential fatty acids [14]. Plant-based diets are also lower in energy density, which could increase the risk of energy malnutrition. These dietary characteristics can potentially affect the growth and development of children. However, data remain very sparse. The majority of research comes from adult studies, and many of the published studies in children are now several decades old and include only vegetarian subjects [15]. Importantly, the last decade has seen a new wave of studies of plant-based diets in children that provide more detailed health outcome data, including biomarkers in vegan children [16,17] 

Of particular concern is that the contemporary increase in plant-based alternatives to animal products is driven by ultra-processed foods, with a markedly different and usually inferior nutritional profile compared to traditional animal- or plant-food alternatives [18]. In order to increase palatability, visual appeal, and shelf-life, they also contain numerous food additives, including added flavors, colors, and emulsifiers [1]. These substances have been shown to modify the human microbiome, although the significance of this is still poorly understood [19]. In their appearance and names, these products mimic meat, dairy, and fish, which may appear to indicate nutritional equivalence. However, data on their nutritional composition are scant, and little is known about their health impacts [1]. Preliminary data indicate lower height in children substituting cow’s milk with plant-based milk [20], no difference in postprandial inflammatory response between plant-substitute versus real meat [21], and a link between chronic food-additive consumption and dysbiosis [22]. Recent data in adults suggest increased cardiovascular and cancer risks and total mortality linked to ultra-processed-type plant-based diets [23].

While plant-based diets are becoming more popular, guidelines of pediatric and dietetic institutions around the world regarding the safety of vegetarian and vegan diets in children’s guides are heterogeneous and conflicting [1,24,25,26,27,28]. Additionally, most countries lack clear nutritional guidelines for vegetarian and vegan children. A recent Italian study found that nearly 9% of Italian infants were weaned to either a vegetarian or vegan diet, but almost half (45.2%) of the parents were unable to obtain appropriate dietary advice from their pediatrician due to doctors’ lack of expertise in the subject [29].

All these issues could affect the safety of plant-based diets among children. The aim of this narrative review is fourfold: (a) to review the available data on secular trends in the consumption of plant-based diets among children in high-income countries, (b) to conduct a systematic review of current data on health outcomes of children on vegetarian and vegan diets, (c) to review the heterogeneity of current dietary advice on plant-based diets for children, and (d) to propose a roadmap of the research questions that urgently need to be addressed in order to provide guidelines on how to safely implement these diets in children.

## 2. Secular Trends in Plant-Based Diets in Children 

Although national survey data remain rare, numerous sources indicate an increased rate of consumption of plant-based diets in high-income countries. In the last 10 years, the number of vegans in Germany increased 15-fold (from 0.1 million in 2012 to 1.58 million people in 2022) [30]. In the 2010 UK National Diet and Nutrition Survey (NDNS), 2% of both adults and children reported being vegetarian, with <1% following a vegan diet [31]. In the most recent 2019 NDNS Rolling Programme, the proportion of people who self-identified as vegetarian or vegan had risen to 4.5%, a 50% increase overall [32]. According to the Euromonitor Health and Nutrition Survey 2020, the percentage of vegans in Western Europe, the US, and Australia varied from 1.8 to 4.5%, and the percentage of vegetarians varied from 2.5 to 6.5%, while the proportion of people reporting that they restrict intake of animal products varied from 28 to 48% [13]. Therefore, in these settings, up to half the population reported at least some degree of reducing their dietary intake of animal foods.

These self-reported dietary patterns are consistent with data on food sales. In the US, the market for plant-based alternatives to animal products grew three times faster than overall food sales in 2021 [18]. In Australia, between 2014 and 2016, the number of vegan food products rose by 92% [33]. The market value of plant-based food sales and unit sales grew by 21% in 13 European countries in just three years, between 2020 and 2022 [34].

It is to be expected that children will follow adults in these trends. According to the 2021 BBC Good Food Nation survey in the UK [35], 8% of British children declared themselves as vegan, 13% as vegetarian, and 21% as those who would like to adopt vegetarianism. Therefore, while data remain scarce, there is evidence of increasing consumption of vegan and plant-based diets in children in high-income populations.

## 3. Associations of Plant-Based Diets with Child Health Outcomes (1980s–2013)

To summarize the literature on this topic, we first describe the previous systematic review by Schuermann et al. [15] and then update it. Schuermann et al. systematically reviewed data on vegetarian and vegan children compared to omnivore children from articles published between the 1980s and 2013 [15]. Case reports and studies from non-industrialized countries were excluded. In their analysis, 24 publications from 16 studies met the inclusion criteria. The studies had heterogeneous methodology, were mostly cross-sectional, usually had small sample sizes, used various definitions of vegetarianism (one study included pescovegetarians (fish-eaters) as vegetarians [36]), often lacked control groups, and tended to include participants from higher social classes. Study samples of vegetarian or vegan children usually included fewer than 100 individuals. The majority of research in that period was conducted on vegetarian children and in Central Europe (*n* = 11 studies), with a large proportion of studies from Poland (*n* = 7) [37,38,39,40,41,42,43,44] and the UK (*n* = 4) [45,46,47]. Eight studies were undertaken in the USA [48,49,50,51,52,53,54,55], most of which were conducted on the Seventh-day Adventist (SDA) population. The majority of studies were published before 2000. Five publications were issued in the 1980s (four in the USA and one in the UK), ten in the 1990s (one in Germany, two in the UK, two in Slovakia, one in Belgium, and four in the USA), and the remaining nine after 2000 (seven in Poland, one in the UK, and one in the USA).

The age range of the children spanned 0 to 18 years; two studies [47,56] included only infants and/or toddlers. Only two studies focused on vegan diets [45,50]. Overall, children following meat-free (vegetarian, vegan, and, from one study only, pescovegetarian) diets were either similar, or somewhat below, the national or omnivore (OM) reference group in terms of height, weight, body mass index (BMI), fat mass, lean mass, and skinfold measures. Vegetarian children demonstrated altered metabolism of bone markers, suggesting impaired bone turnover rate. There were no data on the bone health of vegan children from that research period. Vegetarian children had lower total and LDL cholesterol levels (two studies). In comparison to the omnivore group or physiological reference data, studies from this period suggested either normal micronutrient status or increased risk of iron and vitamin D3 deficiency in vegetarian children. There were no studies on blood micronutrient status in vegans. Schuermann et al. concluded that the existing data at that time did not allow firm conclusions to be drawn on the health benefits or risks of vegetarian or vegan diets in children and adolescents in industrialized countries [15].

## 4. Methods

**Figure 1:**
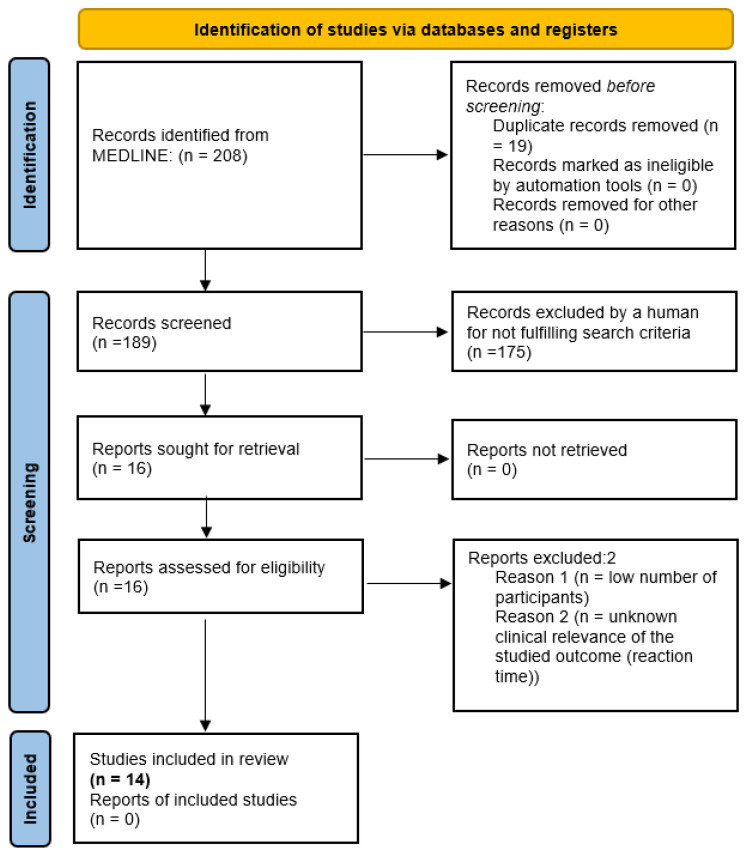
PRISMA 2020 flow diagram for the methodology of this systematic review of studies from high-income countries assessing health outcomes of vegetarian and vegan children from 2013. The bold was used to underscore the final number of the studies.

To update the systematic review, we searched the database MEDLINE (via PudMed) for articles in English, German, or Polish from the 2013–2023 period. Only studies assessing health outcomes were selected, and those reporting only dietary intakes were excluded. For search terms, the following search string was used: (vegetarian OR vegan) AND (infant OR infancy OR child OR toddler OR children OR adolescent OR adolescence OR pregnant OR pregnancy OR breastfeeding OR breastfed). Case studies and studies from non-industrialized countries were not included as the study participants, apart from following plant-based diets, might also have been exposed to inadequate food intake. The Prisma flow diagram for our search results is presented in Figure 1.

One independent reviewer (MD) used the revised Downs and Black Quality Index score system, known to be a reliable and valid tool for assessing bias in observational and randomized studies to assess the quality of individual publications [57]. The quality assessment tool provided an overall score based on four assessed domains: reporting, external validity, internal validity bias, and internal validity confounding. Each domain had an overall total score out of 10, 3, 7, and 6 respectively. Item 27 relating to the statistical power was given a score of 1 when a power analysis had been conducted. Thus, the highest possible score for the checklist was 28. Each paper was assigned a grade of “excellent” (24–28 points), “good” (19–23 points), “fair” (14–18 points), or “poor” (<14 points).

## 5. Associations of Plant-Based Diets with Child Health Outcomes (2013–2023)

Fourteen publications from 13 studies met the same inclusion criteria as those used previously. The majority of the studies were cross-sectional (*n* = 11), but two were longitudinal. 

Most studies assessed vegetarians only; however, six assessed vegans [16,17,58,59,60,61,62]. The sample size ranged from 6 (vegan) [59] to 248 (vegetarian) children [63]. The average sample size of vegan or vegetarian groups was 81 children, with 79 for vegans only and 83 for vegetarians only. In one small study, vegetarians were described as those who also eat fish (*n* = 10) [59]. The age range of the studies spanned 0 to 18 years. Only three studies [16,17,58] assessed the socioeconomic class (SES) of the family, and the majority of participants were from higher SES. Six studies assessed physical activity—five by questionnaires. Only one study, from Poland, [16] matched vegetarian and vegan children with omnivore controls by age, sex, maternal education, and rural/urban location. This was also the only study to obtain objective data on measured physical activity, a potential confounder of associations of child diet group with health outcomes, such as body fat content and cardiovascular risk.

The majority of studies were conducted in central Europe (seven in Poland, two in Germany, and one in the Czech Republic), or other European countries (one in Finland and one in Italy); there was one study from Canada. Unlike in the previous period (before 2017) in which some studies were conducted on Seventh-Day Adventists [64] or collective communities [50], none of the newer studies were conducted specifically on a religious or another belief group. Most of them assessed dietary intake as well as health outcomes.

These new studies contribute new knowledge on biomarkers in vegetarian and, in particular, vegan children and provide the first set of data on bone health in vegan children, more comprehensive bone data in vegetarians, and the first set of data on iodine status and its effect on thyroid function in vegetarian and vegan children. Equally importantly, they provide data on contemporary vegan children. A summary of the design and findings of each study is presented in Table 1.

### 5.1. Body Composition and Anthropometry

All studies measured at least basic anthropometry. Regarding the comparison of vegetarian children with omnivores, most studies did not observe differences in height (HT), weight (WT), BMI, or body composition. However, some studies showed lower values among children on vegetarian and, in particular, vegan diets. More vegetarian (2.4%) than omnivore toddlers (0%) were classified as stunted or wasted in the German VeChi DietStudy [58]. In a longitudinal Canadian study [63], vegetarian children had higher odds of being underweight (BMI z-score < −2), with an odds ratio of 1.87 (95% CI 1.19, 2.96). In a Polish study [70], vegetarian children tended to have lower mean BMI z-scores (−0.18 ± 0.90 vs. 0.19 ± 0.80, *p* = 0.06) compared to OM controls. Most studies did not show differences in body composition between vegetarian and omnivore children; however, a Polish study showed lower gluteofemoral adiposity in vegetarian children using skinfold measurements (thigh girth *z*-score Δ −0.37, 95 CI% −0.69, −0.05) despite similar total fat and lean mass by deuterium dilution [16]. 

On the other hand, most studies assessing vegans reported lower values in WT, HT, BMD, and body composition in comparison to the omnivore control group. In the VeChi DietStudy [58], more vegetarian (3.6%) than omnivore (0%) toddlers were classified as stunted or wasted. An Italian study [60] showed that, compared to omnivores, vegans had lower weight values expressed in grams and percentiles at birth and at 6 and 12 months, along with shorter body length expressed in growth percentiles at 12 months and lower BMI at 6 months. In a German study [17], although no statistical difference in average HT, WT, or mean BMI *z*-score was observed between vegan and omnivore children, there was a tendency for vegan children to have lower values (BMI *z*-score: −0.6 ± 0.9 vs. −0.3 ± 1.0, *p* = 0.15; height: 152 ± 19 vs. 156 ± 20 cm, *p* = 0.49). Despite similar average ages between the three diet groups, vegan children were 4 cm shorter than omnivores. However, the difference was not statistically significant because the analysis incorporated a very wide age range (6–18 years), resulting in very high standard deviations for height, with a range of 19–20 cm. 

In a Polish study, vegan school-aged children were on average 3.2 cm shorter than omnivore children matched for sex, age, and socioeconomic status [16]. This study also showed lower fat indices in vegan children in all body regions measured by skinfolds (suprailiac skinfold *z*-score Δ −0.57, 95% CI −0.97, −0.18; triceps skinfold *z*-score Δ −0.47, 95% CI −0.86, −0.09), hip girth (*z*-score Δ −0.58, 95% CI −0.94, −0.21), and lower fat mass index *z*-score in relation to omnivores (−0.72, 95% CI −1.12, −0.32), but they had similar lean mass, which was measured by deuterium dilution. In a Czech study [62], significantly more vegans were in the ≤3 percentile BMI category (7 out of 79) than in the other two groups (OM: 1 out of 52, vegetarian: 0 out of 69, *p* = 0.03); however, there was no overall difference in BMI, HT, or WT percentiles between groups. In another publication by the same authors [61], vegan children had lower BMI percentiles than omnivore children (vegan: 35.0, IQR 18.2, 63.5; OM: 40.0, IQR 19.5, 55.0, *p* = 0.006) [61].

Overall, it seems that anthropometric markers in vegetarians are comparable to or slightly lower than the reference group, whereas vegan children tend to have lower values of WT, HT, and fat mass than omnivore children. They also have a higher risk of being stunted and wasted; however, the percentage affected is relatively small. 

### 5.2. Bone Health

Four studies since 2013 provided the first set of data on the bone health of vegetarian (*n* = 3) and vegan (*n* = 1) children. These studies, assessing bone mineral content and bone metabolic markers, were all conducted in Poland. In vegetarians, there were either no differences in total-body-less-head (TBLH) bone mineral content (BMC) (729 g ± 226 vs. 76

8 g ± 237, *p* = 0.341 and bone mineral density (BMD) (0.784 g/cm^2^ ± 0.068 vs. 0.799 g/cm^2^ ± 0.080 *p* = 0.700) in comparison to the OM reference group [16] or lower values of the mean spine BMC (57.5 g ± 18.7 vs. 63.0 ± 18.1, *p* = 0.09), lumbar spine (L1–L4) BMD (0.617 g/cm^2^ ± 0.083 vs. 0.645 ± 0.083, *p* = 0.060), mean spine BMD *z*-score (−0.58 ± 0.72 vs. −0.19 ± 0.64,), and mean lumbar spine BMD *z*-score (−0.73 g/cm^2^ ± 0.91 vs. −0.51 ± 0.75, *p* = 0.11) in vegetarian compared to OM children in analyses unadjusted for bone or body size [66]. Vegetarians had significantly higher levels of the blood biochemical markers BALP (130.7 U/L ± 39.9 vs. 108.4 ± 37.1, *p* = 0.002) and CTX-I (1.976 ± 0.538 vs. 1.749 ± 0.526, *p* = 0.027), which were interpreted as a higher rate of bone turnover in vegetarian compared to OM children [66]. In another study from the same group, bone and body size unadjusted TBLH-BMD *z*-score (but not absolute values of TBLH BMD) were lower in vegetarians in comparison to omnivores (−0.583 ± 0.718 vs. −0.194 ± 0.642, *p* = 0.009). A similar trend was observed for L2–L4 spine BMD (0.621 ± 0.089 vs. 0.649 ± 0.096, *p* = 0.046) and BMD L2–L4 *z*-score (−0.877 ± 0.858 vs. −0.496 ± 0.791, *p* = 0.019). Vegetarian children also had higher PTH (40.8 pg/ML, 95% CI 29.5, 57.2, vs. 32.1, 95% CI 23.1, 42.5, *p* = 0.015). In another study from Poland, there was no difference in TBLH BMC, L2–L4 BMC, or bone mineral apparent density (BMAD) *z*-score in 5–10-year-old vegetarian children in comparison to omnivores matched for age, sex, and SES status [16]. Unlike in the previous Polish studies investigating the bone health of vegetarian children, this study adjusted BMC values for body size and bone area. BMAD adjusts BMC for calculated bone volume rather than bone area. The only study to evaluate vegan diets [16] showed lower TBLH (Δ −3.7%; 95% CI −7.0, −0.4) and lumbar spine (L2–L4) (Δ −5.6%; 95% CI (−10.6, −0.5) BMC in Polish vegan compared to OM children aged 5–10 years old in analyses adjusted for bone size in order to take into account the lower body size of the vegans. This was confirmed by another approach that corrected for bone size, whereby the BMAD *z*-score and percentile were significantly lower for vegans only.

Overall, the available data show a trend of lower BMC and BMD values in vegetarian children; however, significant differences were only observed in bone and body size unadjusted analyses. Vegan children have lower values of BMC, and the difference attenuates but persists after adjusting for bone and body size. Vegetarian children have altered bone metabolism markers. There are no data on bone marker metabolism in vegan children. 

### 5.3. Nutritional Biomarkers

Seven publications (from sux studies) assessed nutritional biomarkers, and five of these addressed vegan as well as vegetarian children. 

Overall, vegetarian children had lower levels of ferritin in some studies (median levels in ng/mL 21.0, IQR 15.0–29.0 vs. 27.0 IQR, 21.6, 48.7, *p* = 0.003) [65]; 29, IQR 20–39 vs. 38, IQR 26–52, *p* = 0.0312) [17] but not in all studies [16,63]. There was no significant difference in hemoglobin (Hb) concentrations [16,17], and a Polish study [65] showed no difference in transferrin levels between vegetarian and omnivore children, while other hematologic parameters and serum iron were within the reference range in the vegetarian children. Vegans, on the other hand, had lower indices of ferritin in two studies [16,17] (Δ in relation to OM −25%, 95% CI −44.0–−5.0), with a median of 29 ng/mL in vegans (IQR 22–42 vs. 38 in OM, IQR 26–52), lower levels of Hb (Δ in g/dL −0.37, 95% CI −0.69, −0.05) and erythrocytes (Δ in M/μL −0.23, 95% CI −0.33, −0.12) in one study [16], but similar Hb concentrations compared to the OM group in another study that assessed iron metabolism [17]. 

Vegetarians had lower levels of vitamin B12 and holotranscobalamin (holoTC) and higher levels of other biomarkers, suggesting insufficiency of vitamin B12 (methyl malonic acid (MMA) and homocysteine (Hs)) if the children did not supplement with vitamin B12 (serum vit. B12 difference in pmol/L −90.9, 95% CI −156.7, −25.1) [16,17,62]. These differences disappeared in supplemented vegetarian children [16,62]. Similar trends were reported for vegan children; however, the data indicate that vitamin B12 deficiency in unsupplemented vegans tends to be more pronounced (serum vit. B12 difference in pmol/L −217.6, 95% CI −305.7, −129.5) [16]. Moreover, a Czech study [62] reported vitamin B12 hypervitaminosis in over-supplementing vegans and vegetarians, the significance of which is unknown. 

**Figure 2:**
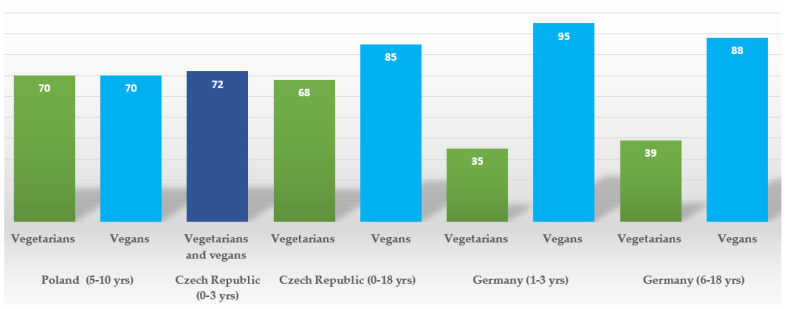
Vitamin B12 supplementation rates (%) among vegetarian and vegan children by country.

Across different studies, the rates of vit B12 supplementation were as follows: 39% and 88% in German vegetarians and vegans, respectively, aged 6–18 years old [17]; 35% and 95%, respectively, among German vegetarian and vegan toddlers aged 1–3 years old [72]; 70% in both vegetarian and vegan children aged 5–10 years old in Poland [16]; 68% and 85% of vegetarian and vegan Czech children, respectively, aged 0–18 years old; and 72% in a subgroup of both vegetarian and vegan Czech infants, respectively [62]. Based on these data, substantial proportions of children of vegan and vegetarian children across European populations do not receive vitamin B12 supplements, which can result in serious hematological, metabolic, and neurological complications [73,74,75]. The available evidence regarding the rates of supplementation of vitamin B12 in vegetarian and vegan children is summarized in Figure 2.

Other nutritional biomarkers measured were fat-soluble vitamins A, D, and DHA, which were markedly lower among vegan children in a small study of vegan and vegetarian Finnish preschoolers [59]. A study that measured serum vitamin B2, 25-hydroxy vitamin D (25(OH)D), and folate among German children aged 6–18 years old found that only folate differed between the groups, being highest in vegan children [17]. All dietary groups in this study had a high prevalence (>30%) of 25(OH)D and vitamin B2 concentrations below reference values; however, the percentage for vitamin B2 tended to be higher in vegan and/or vegetarian than OM children (vegetarian: 50%, vegan: 54%, and omnivore: 37%).

In a Polish study on school-aged children, 25(OH)D was lower in both unsupplemented vegetarian (Δ in nmol/L −7.1, 95% CI −13.8, −0.3) and vegan (Δ in nmol/L −13.3, 95% CI −20.3, −6.2) children compared to OM controls, with the difference being most pronounced in vegans [16]. 

A Czech study was the first to measure iodine in spot urine (UIC) in vegan and vegetarian infants and children [61]. The UIC values were highest in the OM control group, followed by vegetarian, with the lowest levels among the vegan children. Forty-two percent of vegans, 35% of vegetarians, and 20% of omnivore children (*p* = 0.06) met the criteria for iodine deficiency (i.e., UIC < 100 µg/L). This may have health consequences as the same study found negative effects on thyroid function. Higher levels of fT4 were found in vegans than in the OM group (*p* < 0.001), and a higher presence of anti-thyroglobulin antibodies (AhTGc) in vegetarians (18.2%) and vegans (35.0%) than in the OM group (2.1%) (*p* < 0.001) was detected. It was hypothesized that this was due to lower iodine intake among vegetarian and vegan children. 

Serum amino acid levels (valine, lysine, leucine, and isoleucine) were lower in Polish vegetarian children compared to OM controls [71], while branched-chain amino acids (valine, leucine, and isoleucine) were lower in preschool Finish vegan than in omnivores in a study by Hovinen et al. 2023 [59]. However, the health implications of these differences remain unclear.

Overall, both vegetarian and vegan children develop signs of vitamin B12 deficiency if each diet is not supplemented with this nutrient. The average percentage of studied vegetarian and vegan children whose diet is not supplemented with vit. B12 is ca. 30%. Both vegetarian and vegan children have lower levels of iron stores (ferritin); however, other iron metabolism markers are usually similar to or slightly lower than the reference group. Both vegetarian and especially vegan children are at risk of vitamin D deficiency if they do not supplement and can have inadequate iodine intake. The suboptimal iodine status of vegetarian and vegan children might affect thyroid function. Vegans might have lower levels of other, especially fat-soluble, nutrients; however, more studies are needed to confirm the preliminary findings. 

### 5.4. Cardiometabolic Risk Factors

In adults, plant-based diets have been associated with lower NCD risk [76]; however, whether such benefits are evident in childhood has only recently been addressed. 

Polish 5–10-year-old vegetarian children had significantly higher ratios of anti-inflammatory to pro-inflammatory adipokines compared to omnivore children [67]. Another study from Poland, assessing 5–10-year-old vegetarians and SES-matched omnivore children found lower total cholesterol (TC) (Δ in mg/dL −11.5, 95% CI −22.4, −0.6) and HDL-cholesterol (HDL-C) (Δ in mg/dL −6.5, 95% CI −11.1, −1.8) in vegetarians; however, they also had higher serum fasting VLDL-cholesterol (Δ in % 14.0, 95% CI 1.0, −28.0), triglyceride (Δ in % 19.0, 95% CI 5.0, 33.0), and glucose levels (Δ in mg/dL 3.1, 95% CI 0.9, 5.4), suggesting less favorable cardiometabolic risk profile comparison to the meat-eating reference group. There were no differences in fasting insulin levels, high sensitivity C-reactive protein (hs-CRP), or ultrasonography-assessed carotid intima-media thickness (cIMT) between vegetarian and omnivore children [16]. German 6–18-year-old vegetarians did not differ from the OM reference group in any of the lipid fractions measured: TC, non-HDL-C, HDL-C, LDL-cholesterol (LDL-C), or triglycerides (TG) [17]. No association of serum lipids (non-HDL-C, TC, LDL-C HDL-C, and TG) with a vegetarian diet was found in a longitudinal study of Canadian vegetarians [63]. Most recently, Polish vegetarians (2–10 years old) had significantly lower median values of total oxidant capacity, oxidative stress index, and CRP and higher total antioxidant capacity [67].

More pronounced differences in cardiometabolic risk factors were reported in vegans. All fractions of blood lipid levels were significantly lower in vegan than omnivore Finish preschoolers [59]. Six-to-eighteen-year-old German vegans had the lowest non-HDL-C concentration (median in mg/dL 78, IQR 63, 94 vs. 96, IQR 73, 113 in OM, *p* = 0.0010) and LDL-C (median in mg/dL 68, IQR 57, 84 vs. 90, IQR 70, 106 in OM, *p* = 0.0010) in comparison to omnivores; however, their HDL-C levels did not differ from other groups [17]. 

Polish 5–10-year-old vegans had significantly lower TC (Δ in mg/dL –35.6, 95% CI –48.3, −22.9), LDL-C (Δ −24.0, 95% CI −35.2, −12.9), and HDL-C (Δ −12.2, 95% CI −17.3, −7.1) fractions, along with lower hs-CRP (Δ in % −81, 95% CI −123.0, −39.0) in comparison to the matched omnivore group [16]. The prevalence of low HDL-C levels was highest and the prevalence of high LDL-C levels was lowest among vegans in this study. There was, however, no difference in fasting serum glucose, insulin levels, or cIMT in comparison to omnivores.

Overall, evidence shows that children consuming a vegan diet have a more favorable lipid profile than OM children; however, the results for a vegetarian diet were inconsistent and varied by outcome.

### 5.5. Assessment of Bias

**Figure 3:**
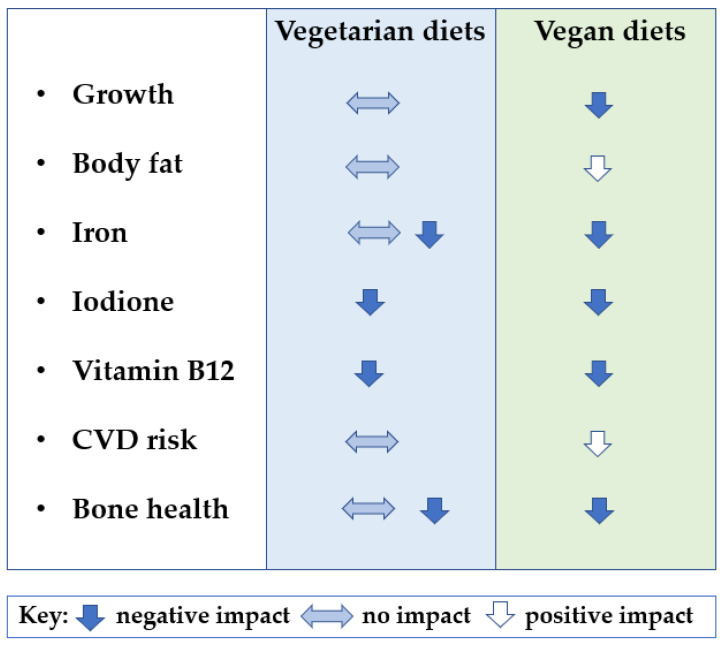
Summary of impacts of vegetarian and vegan diets on a range of children’s health outcomes based on the evidence listed in Table 1.

The assessments of bias for the articles identified in this review are tabulated in Table S1. Ten studies were rated as fair and four as poor, with none being rated good or excellent. Very few studies provided data on vegan or vegetarian children that were representative of these populations. There is currently, therefore, relatively weak evidence regarding the effects of vegetarian and vegan diets on children’s health. Despite these limitations in the quality of evidence, the existing data show relative consistency in the associations of both types of diet with health outcomes, as illustrated in Figure 3.

## 6. Conflicting Position Statements of Medical and Nutritional Institutions around the World

While plant-based diets are increasingly consumed by children and have implications for their health, as reviewed above, there is a notable lack of universal agreement regarding the safety of vegetarian, and, in particular, vegan diets for children around the world. This was recently highlighted, in particular, for vegan children [77]; here, we summarize the scenario for both vegan and vegetarian diets.

Positive position statements come from the nutrition and dietetic institutions of the USA, the UK, and Italy. The US American Academy of Nutrition and Dietetics (formerly known as the American Dietetic Association) has been advocating the safety of well-balanced vegetarian and vegan diets for all life stages, including infancy, childhood, and adolescence, for the last 30 years, in their position statements, beginning in 1993 [78], followed by updates in 2009 [79] and 2016 [25]. Similarly, the British Dietetic Association (BDA) considers that a well-planned vegan diet can support healthy living in people of all ages [24]. The Italian Society of Nutrition states that a vegetarian diet (which is defined in the position paper as either a lacto-ovo vegetarian or vegan diet), providing that it includes a variety of foods and vitamin B12 supplements, provides adequate nutrition for people of all ages [80].

The majority of other pediatric and nutrition institutions from around the world express a more or less cautionary approach, ranging from recommendations to exercise caution or to avoid veganism in the early stages of life to statements declaring an outright lack of support for this type of nutrition for children, with an emphasis on the high risk of impairments of growth and development. 

A joint statement of Health Canada, the Canadian Paediatric Society, Dietitians of Canada, and the Breastfeeding Committee for Canada from 2014 emphasizes the need for professional advice for vegetarian infants and informs that, with careful planning, vegetarian diets can meet all the nutritional requirements of a growing child, provided the diet contains milk and eggs. It stresses a higher risk from vegan diets and the need for professional advice for vegan infants [81]. Earlier in 2010, the Canadian Paediatric Society warned of significant medical consequences of inappropriately planned diets in children and the lack of data regarding the development, growth patterns, and nutrition intakes of children on plant-based diets [82]. 

The Nutrition Committee of the Argentine Society of Pediatrics emphasizes that parents need to be aware of the risks associated with more restrictive diets, including vegetarian diets in childhood, stresses the need for professional nutritional guidance, and concludes that, without careful planning, those diets cannot be safely applied in childhood [83]. 

The Committee on Nutrition and Breastfeeding of the Spanish Pediatric Association emphasizes the need to carefully plan vegetarian and vegan diets; however, it does not recommend veganism, but rather an omnivore diet, or at least an ovo-lacto-vegetarian diet for infants and young children [84].

The 2016 position statement of the German Nutrition Society (the DGA, Deutsche Gesellschaft fuer Ernaehrung) reads “(…) veganism is not recommended by DGE for (…) infants, children and adolescents” [85]. In the 2020 update to that statement, the DGE upheld its position and does not recommend veganism for children and adolescents; however, it added that careful choice of foods and supplements, especially vitamin B12, can lead to adequate provision of nutrients, although this statement does not define any life stage [86].

The French Pediatric Hepatology, Gastroenterology, and Nutrition Group (GFHGNP) states that a vegan diet does not provide all micronutrients and exposes children to inevitable nutritional deficiencies, which can have serious consequences for health, especially when introduced at an early stage [87]. Therefore, GFHGNP does not recommend veganism in infants, children, and adolescents.

The Belgian Royal Academy of Medicine does not recommend veganism in small children and describes it as potentially destabilizing growth [26]. 

The Committee of Human Nutrition Science of the Polish Academy of Sciences states in their 2019 position statement that veganism and other forms of vegetarianism excluding meat should not be applied in infants and young children as they may adversely affect their development [28]. In contrast, the position statement of the Polish Society of Pediatric Gastroenterology, Hepatology, and Nutrition from 2021 stresses that vegetarian, and, in particular, vegan infants and young children require supplementation and regular consultations with nutrition specialists and that parents of vegan children should be aware that lack of supplementation can have serious consequences for the child’s health or even life [88].

According to the recently published opinion of the Norwegian Nutrition Council (NNC), vegetarian and vegan diets may be suitable at all life stages, including children, if they are well planned and accompanied by supplementation of vitamins and minerals [89]. To back its statement, the NNC cites the most recent US Academy of Nutrition and Dietetics position paper [25]. 

Finally, the European Society for Paediatric Gastroenterology, Hepatology, and Nutrition and the German Nutrition Society reflects the tone of the majority of European position statements, stating that, in small children, a vegan diet can meet nutrient requirements but that ‘the risks of failing to follow advice are severe’, and stressing the need for regular medical and expert dietetic supervision of vegan children [90]. 

In summary, the majority of the European statements express concerns about the risks and need for constant medical and dietetic supervision. However, as Kiely pointed out, over 40% of EU countries have general-practitioner-led care with a median of 4 months of pediatric training and inadequate nutrition education in medical schools. Hence, concerns that without specialist pediatrician and dietetic follow-up, growth and development delays due to an unbalanced vegan or vegetarian diet might not be detected in time, are valid [91]. 

The apparent contradictions between US and European statements were highlighted by Pawlak [92], who stressed the difference in the clarity of advice provided with regard to supplementation, dietary strategies for vegans, the need for medical/dietetic supervision required, and the responsibilities of parents and suggested that the efficacy of these statements should be tested in trials. 

Despite the universally available evidence, different organizations are generating contrasting recommendations, with the differences especially marked for vegan diets but also evident for vegetarian diets. It remains unclear if these different approaches are based wholly on children’s health criteria or whether they are also influenced by other factors such as planetary or animal welfare concerns.

## 7. Roadmap for Future Research

While the last decade has seen a new wave of research on the health impacts of plant-based diets in children, a number of outstanding questions remain and would benefit from further research. These relate, in particular, to whether health benefits and risks vary by stage of development, the impact of supplementation of bone-supporting nutrients on bone health in vegan children, the consequences of consuming new plant-based ultra-processed foods, and whether health effects seen in children will have longer-term impacts on adult health. We summarize a number of questions for both research and urgent practice regulation in Table 2.

First, health professionals, especially doctors who provide regular medical care for children, depending on the country’s medical models (general practitioners and/or pediatricians), should receive basic training to enable them to provide families of those children with fundamental nutritional and supplementation advice. This would at least prevent the most severe consequences of inadequate application of those diets in children. Second, dietary and supplementation guidelines tailored to different stages of children’s development but also catering to regional food and cultural habits should be introduced in each country (as illustrated in Figure 2). This would facilitate both doctors and dieticians to give more detailed dietary counseling in the context of time constraints of medical consultation and/or lack of advanced nutritional training. Additionally, it would allow daycares and educational institutions (creches and schools) to provide appropriately balanced meals for vegetarians and vegans.

Lastly, there is a need for clear regulations of ultra-processed meat and dairy alternatives regarding their suitability for children and transparency regarding their nutritional equivalence (or lack of it) to their animal-based counterparts.

## 8. Conclusions

Children are increasingly consuming plant-based diets and the available evidence indicates that this can have implications for their growth and body composition, bone health, nutritional biochemistry, and cardio-metabolic risk. The findings of cardiometabolic benefits have major implications for efforts to reduce non-communicable disease risk through the life course; however, children consuming plant-based diets, in particular, vegan children, may also be at risk of nutritional deficiencies with long-term effects. Since peak bone mass is attained in early adulthood, low levels of bone density in childhood merit particular attention. A third of those children might be at risk of vitamin B12 deficiency, which can result in developmental impairment. The heterogeneity of advice by different national and international organizations indicates that a process of harmonization is warranted, which requires expansion of the evidence base. We highlighted a number of areas where further research should be prioritized. Additionally, we identified a number of practice recommendations that should be urgently introduced at national and international levels to facilitate/improve the safety of the application of vegan and vegetarian diets in children.

## Figures and Tables

**Table 1:** Summary of the health outcome data of vegetarian and vegan children (0–18 yrs) compared to omnivore children, published between 2013 and 2023.

Authors, Year, Place	Study Characteristics	Participants	Dietary Data Collection	Health Outcomes Measured	Results
Ambroszkiewicz et al. 2017. The Institute of Mother and Child (IMC) in Warsaw, Poland [65].	Cross-sectional data collected 2015–2016 from patients seeking dietary counseling.	43 vegetarian (VG) and 46 omnivore (OM) prepubertal (4.5–9.0 years). No data on socioeconomic status (SES) or physical activity (PA).	3-day food diary data on average daily energy, protein, fat, carbohydrates, and dietary iron and vitamin intakes.	Serum hemoglobin (Hb), red blood cells, mean corpuscular volume, iron, ferritin, transferrin, C-reactive protein (CRP), hepcidin (bioactive heptidin-25 molecule), and soluble transferrin receptor concentration (sTfR), weight (WT), and height (HT)	Lower ferritin and hepcidin concentrations in VG vs OM sTfR concentrations significantly higher in VG No differences in serum transferrin No differences in WT, HT, or BMI
Ambroszkiewicz et al. 2018. The IMC, Warsaw, Poland [66].	Cross-sectional data collected 2014–2017 from a group of patients seeking dietary counseling.	70 children on VG diet from birth and 60 OM children (5–10 years). No data on SES. PA study inclusion criterion: more than 2h of activity per week.	3-day food diary; data on average daily energy, protein, fat, carbohydrates, and dietary mineral and vitamin intakes.	Bone mineral content (BMC) and density (BMD) in total body (tBMD) and lumbar spine (BMD L1–L4), lean and fat mass by dual-energy X-ray absorptiometry (DXA), bone alkaline phosphatase (BALP), C-terminal telopeptide of type I collagen (CTX-I), osteoprotegerin, nuclear factor κB ligand, sclerostin, Dickkopf-related protein 1, 25-hydroxyvitamin D, 25(OH)D, parathormone (PTH), HT, and WT.	No significant differences in body composition, HT, BMI z-scores, BMC, BMD, and 25(OH)D between VG and OM children; however, there was a trend for spine BMC and BMD of VG to be lower; VG had significantly higher levels of BALP and CTX-I (interpreted as a higher rate of bone turnover markers) and higher median levels of PTH than OM.
Ambroszkiewicz et al. 2018. The IMC, Warsaw, Poland [67].	Cross-sectional data collected 2017–2018 from patients seeking dietary counseling.	62 children on a VG diet from birth and 55 OM children (5–10 years). No data on SES. PA was assessed by a questionnaire.	3-day food diary; data on average daily energy, protein, fat, carbohydrates, and dietary fiber intake.	Serum concentrations of adipokines: leptin, soluble leptin receptor (sOB-R), adiponectin, resistin, visfatin, vaspin, and omentin; fat mass and fat-free mass by DXA; HT and WT.	No differences in WT, HT, BMI, or body composition between groups. VG had lower leptin/sOB-R ratio and lower serum resistin; other adipokines did not differ between both groups; VG had significantly higher ratios of anti-inflammatory to pro-inflammatory adipokines, adiponectin/leptin, and omentin/leptin.
Ambroszkiewicz et al. 2019. The IMC, Warsaw, Poland [68].	Cross-sectional data collected 2014–2016 from patients seeking dietary counseling.	53 children on a VG diet, 53 OM children (5–10 years). No data on SES. PA was assessed by a questionnaire.	3-day food diary; data on average daily energy, protein, fat, carbohydrates, and dietary mineral and vitamin intakes.	WT, HT; body composition and BMD by DXA, 25(OH)D, and PTH, serum carboxy-terminal propeptide of type I collagen (CICP), total osteocalcin and its carboxylated and undercarboxylated forms, CTX-I, and leptin and adiponectin levels.	No differences in HT, WT, BMI z-scores, or body composition between VG and OM. Mean total BMD z-score and lumbar spine BMD z-score lower in VG; however, absolute bone mineral density did not differ; serum leptin level 2-fold lower in VG, reflecting lower body fat; VG had higher PTH and CTX and similar levels of adiponectin, osteocalcin, CICP, and 25(OH)D.
Weder et al. 2019. The VeChi DietStudy, Germany [58].	Cross-sectional data collected 2016–2018 children throughout Germany. OM children were partially recruited from the DONALD study [69].	127 VG, 139 vegan (VN), 164 OM children (1–3 years). SES and urbanicity data collected, PA assessed by a questionnaire.	3-day weighed dietary records; breast milk intakes were estimated [69]. Energy, macronutrients, and fiber intakes calculated.	Data from parents or WT and HT assessed during medical check-up.	Anthropometrics did not differ between diet groups and indicated normal growth. However, more VN (3.6%) and VG (2.4%) than OM children (0%) classified as stunted or wasted.
Hovinen et al. 2021. Municipal daycare lefts, Helsinki, Finland [59].	Cross-sectional data collected 2017 from 20 municipal daycare lefts offering vegan meals in Helsinki.	6 VN (vegan from birth), 10 VG and 24 OM children (1–7 years). No data on PA or SES.	Children were consuming nutritionist-planned diets in daycare lefts, designed to meet nutritional recommendations.	WT, HT, mid-upper arm circumference (MUAC); serum amino acids, vitamin A, 25(OH)D, DHA, and other micronutrients; total cholesterol, HDL-C, LDL-C, endogenous hepatic cholesterol biosynthesis markers, and bile acid biosynthesis markers.	No difference between diet groups in HT, BMI, or MUAC. All fractions of blood lipid levels significantly lower in VN than in OM. Biomarkers for amino acids, fat-soluble vitamins A, D, and DHA markedly lower in VN. The bile acid biosynthesis pathway differed between VN and OM; VN had bile acid pathway similar to profile of fasting children.
Ferrara et al. 2021. Italy [60].	Longitudinal study of infants born to mothers on VN, VG, and OM diets; data collected 2017–2018. Participants recruited via Campus Bio-Medico University Hospital, Romand vegetarian societies.	21 infants each from VN, VG and OM pregnancies.	Food frequency questionnaire to classify mothers according to appropriate dietary pattern.	Weight and length at birth, 6 months, and 12 months.	VN infants had lower WT at birth, 6 and 12 months than OM. VN infants had lower length at 12 months and lower BMI at 6 months than OM. No differences between OM and VG.
Alexy et al. 2021. The VeChi Youth Study, Germany [17].	Cross-sectional data in 2016–2018 from VG, VN, and OM children throughout Germany.	149 VG, 115 VN, and 137 OM children (6–18 years) SES data collected, PA assessed by a questionnaire.	3-day weighed dietary records; energy, macronutrients, selected micronutrients, and supplement use calculated.	HT, WT; blood parameters: Hb, vitamin B2, and folate; ferritin, 25(OH)D, holotranscobalamin (holoTC), methylmalonic acid (MMA), triglycerides TG), and total, LDL, and HDL cholesterol.	No difference in average HT, WT, and BMI z scores; however, generally lower values in VN; no significant difference in median Hb, vitamin B2, 25(OH)D, HDL-C, and TG concentrations. VN had higher folate concentrations than VG; VN and VG had lower ferritin concentrations than OM; VG, but not VN, had lower holoTC and higher MMA than OM, reflecting high (88%) vit. B12 supplementation prevalence in VN, but not VG (39%). VN had lowest non-HDL-C and LDL-C concentrations.
Desmond et al. The Children’s Memorial Health Institute, Warsaw, Poland [16].	Cross-sectional data collected 2014–2016; recruited from advertisements.	63 VG, 52 VN, and 72 OM, (5–10 years). SES and urbanicity data collected. PA data were collected by accelerometry.	4-day food diary and animal product consumption screener; energy, macronutrient intakes, most micronutrient intakes, and supplemental practices were ascertained.	HT, WT, body girths; skinfolds; Body composition (BC) by deuterium dilution and DXA; cardiovascular risk factors: serum total cholesterol (TC), HDL cholesterol (HDL-C), LDL cholesterol (LDL-C), VLDL cholesterol (VLDL-C), triglycerides (TG), high-sensitivity C-reactive protein (hs-CRP), fasting glucose, IGF-1, IGFBP-3; carotid intima-media thickness (cIMT) by ultrasonography; micronutrient status by complete blood count, including mean corpuscular volume (MCV), serum ferritin, vitamin B12, homocysteine (hcys), 25(OH)D; bone health was assessed by DXA (bone mineral content in total body and spine (L1-L4) adjusted by body size) and by calculating bone apparent mineral density (BMAD).	VG had lower gluteofemoral adiposity, similar total fat and lean mass. VN had lower fat indices in all regions but similar lean mass. VG and VN had lower total and L1-L4 BMC; the difference persisted only in VN after adjusting for bone size. VG had lower TC, HDL-C, serum B12, and 25(OH)D without supplementation and higher glucose, VLDL, and TG. VN were shorter and had lower TC, LDL-C, HDL-C, hs-CRP, iron status, serum B12, and 25(OH)D without supplementation but higher hcys and MCV. Vitamin B12 deficiency, iron deficiency, anemia, low ferritin, and low HDL were more prevalent in vegans. Supplementation resolved low B-12 and 25(OH)D concentrations in both groups.
Světnička et al. 2022. The Czech Vegan Children Study (CAROTS), Czech Republic [62].	Cross-sectional data collected 2019–2021; recruited via GPs and advertisements.	200 children: 79 VG, 69 VN, and 52 OM; aged 0 to 18 years old. No data on PA or SES were collected.	3-day weighed dietary records; energy and macro- and micronutrient intakes, along with supplemental practices were ascertained; breast milk intakes were estimated from mothers’ registrations and general recommendations for breast milk intake.	WT, HT; blood concentration of holoTC, cyanocobalamin (B12), folate, hcys, MCV, and Hb.	No difference in BMI, HT, and WT percentiles between groups; VN tended to have lower median BMI and weight percentile; no significant differences in levels of holoTC, folate, hcys, or MCV; 1 VG and 2 VN children were identified as being B12-deficient; however, 83% of vegan children and 70% vegetarians supplemented with vit. B12. A total of 35 VG (44%), 28 VN (40%), and 9 OM children had vitamin B12 hypervitaminosis, related to over-supplementation. Significant differences in B12, holoTC, and hcys levels of supplemented vs. non-supplemented VG/VN children.
Světnička et al. 2023. The Czech Republic [61].	Cross-sectional data collected 2019–2021; recruited via GPs and advertisements.	91 VG, 75 VN and 52 OM children (0 to 18 years). No data on PA or SES were collected.	3-day weighed dietary records to evaluate iodine intake; the use of iodine supplements and their dosages and frequencies were assessed by questionnaire; breast milk intakes were estimated from mothers’ registrations and general recommendations for breast milk intake.	WT, HT; serum TSH, fT4, and fT3; thyroglobulin; levels of anti-thyroid peroxidase antibody (ATPOc) and anti-thyroglobulin antibodies; (AhTGc) concentration of iodine in spot urine (UIC).	No differences in WT and HT percentiles between groups, but lower BMI z-scores in VN. No differences in TSH levels, fT3, thyroglobulin, or ATPOc between groups; higher levels of fT4 in VN compared to OM. Presence of AhTGc more common in VG and VN than OM group. UIC highest in OM group. A total of 31 VN, 31 VG, and 10 OM children met criteria for iodine deficiency (i.e., UIC < 100 µg/l).
Elliott et al. 2023. The TARGet Kids! cohort study. Canada [63].	Longitudinal cohort study. Data collected repeatedly between 2008 and 2019 during scheduled health visits in primary care practices.	8907 children, including 248 VG children at baseline (6 months to 8 years). No data on PA or SES were collected.	The dietary group was assessed by parental self-declaration of the child being on a vegetarian or vegan diet. Both were classified as vegetarian (VG).	HT, WT, serum ferritin, 25(OH)D, and serum lipids (non-HDL-C, TC, LDL-C, HDL-C, and TG).	No difference in BMI z-score, height- z-score, serum ferritin, 25(OH)D, or serum lipids. VG had higher odds of underweight (BMI z-score < −2); no association of diet with overweight or obesity.
Rowicka et al. 2023 The IMC, Warsaw, Poland [70].	Cross-sectional data collected 2020–2021 from patients seeking dietary counseling	32 VG and 40 OM children (2–10 years). No data on SES were collected.	3-day food diary; average daily energy intake, percentage of energy from protein, fat, and carbohydrates, and vitamin intakes determined.	HT, WT; serum CRP, calprotectin, total oxidant capacity (TOC), total antioxidant capacity (TAC), reduced (GSH), and oxidized (GSSG) glutathione; the oxidative stress index (OSI) and the GSH/GSSG ratio were calculated.	No differences in BMI between groups. VG had lower median values of TOC, GSH, GSSG, and CRP and higher TAC compared to OM. OSI significantly lower in VG.
Ambroszkiewicz et al. 2023. The IMC, Warsaw, Poland [71].	Cross-sectional data collected 2020–2021 from patients seeking dietary counseling.	51 VG and 25 OM children (4–9 years old). No data on SES were collected, PA was assessed by questionnaire.	3-day food diary; average daily dietary energy, protein, fiber, calcium, phosphorus, magnesium, vitamin D, and amino acid intakes were assessed in 61 (80%) of the studied children.	WT, HT; serum amino acids, 25(OH)D, PTH, bone metabolism markers (osteocalcin, CTX-I, osteoprotegerin, IGF-I), albumin, and prealbumin.	No difference in BMI between VG and OM; serum concentrations of 4 amino acids (valine, lysine, leucine, and isoleucine) 10–15% lower in VG than in OM; serum differences in amino acid levels less marked than dietary intake differences. VG had lower (but still normal) serum albumin and higher CTX-I (bone resorption marker) than OM. No difference in other bone metabolism markers or PTH levels between groups.

Abbreviations: 25(OH)D: 25 hydroxy vitamin D, AhTGc: anti-thyroglobulin antibodies, ATPOc: anti-thyroid peroxidase antibody, BC: body composition, BMAD: bone apparent mineral density, BMC: bone mineral content, BMD: bone mineral density, CRP: c-reactive protein, DXA: dual-energy X-ray absorptiometry, Hb: hemoglobin, hcys: homocysteine, holoTC: holotranscobalamin, HT: height, MMA: methylmalonic acid, MUAC: mid-upper arm circumference, OM: omnivore, OSI: the oxidative stress index, PA: physical activity (PA), PTH: parathormone, SES: socioeconomic status, sOB-R: soluble leptin receptor, sTfR: soluble transferrin receptor concentration, TAC: total antioxidant capacity, TOC: total oxidant capacity, UIC: concentration of iodine in spot urine, VG: vegetarian, VN: vegan, WT: weight.

**Table 2:** Summary of the outstanding research questions and urgent practice recommendations for scientists and clinicians working with vegetarian and vegan children.

Research Questions	Clinical Practice Recommendations
Do the benefits and risks of vegetarian and vegan children vary by stage of development? Good quality studies in different age groups are needed.	Introduction of country-specific training for medical practitioners on the appropriate pediatric and nutritional care for vegan and vegetarian children and their families.
Does supplementing vegan children with bone-supporting nutrients improve their bone health?	Introduction of national dietary and supplementation guidelines for vegans and vegetarians tailored to children’s stages of development and regional food preferences.
What are the long-term health effects of consuming ultra-processed meat and dairy alternatives?	Regulation of ultra-processed meat and dairy alternatives for their suitability for children and transparency about their nutritional equivalence to their animal-based counterparts.

## Data Availability

The original contributions presented in this study are included in this article/Supplementary Materials; further inquiries can be directed to the corresponding author.

## Supplementary Materials

The following supporting information can be downloaded at: https://www.mdpi.com/article/10.3390/nu16050723/s1, Table S1: The assessments of bias for the studies included in the systematic review.
